# Supplementary material for: MSK1 Downstream Signaling Contributes to Inflammatory Pain in the Superficial Spinal Dorsal Horn
Source: Int J Mol Sci. 2025 Dec 18;26(24):12177. doi: 10.3390/ijms262412177 (PMC12733777; doi:10.3390/ijms262412177)
Supplement: Supplementary file 1 [file ijms-26-12177-s001.zip › ijms-4019684-supplementary.pdf]

Supplementary Figures

Supplementary Figure S1

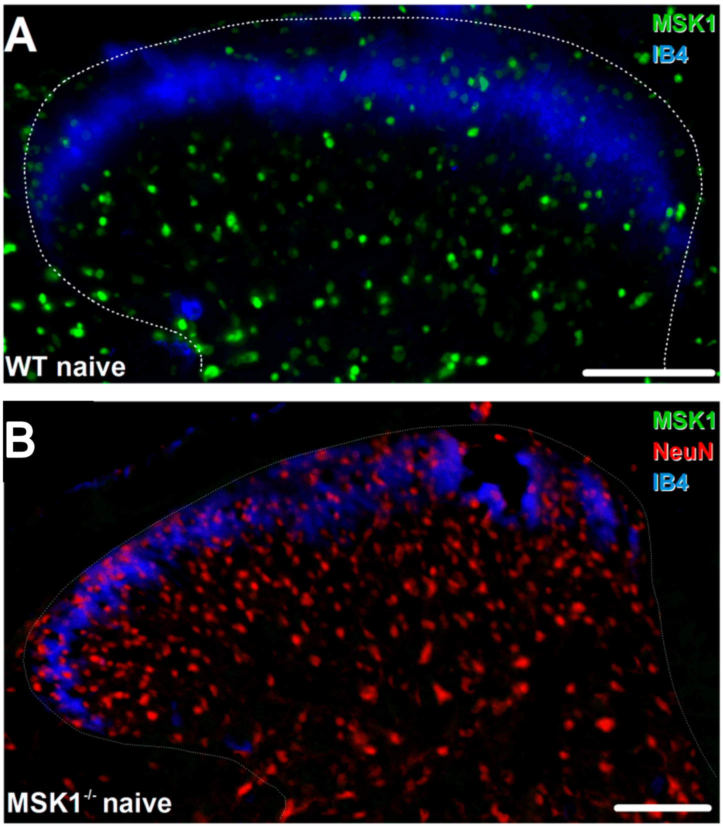

MSK1 expressed wild type but not in MSK1$^{-/-}$ mice's spinal dorsal horn.

(A) Microphotograph of a wild type mouse's the spinal dorsal horn depicting MSK1-expressing nuclei. **(B)** Microscopic image of a MSK1$^{-/-}$ mouse's spinal dorsal horn showing no immunoreactivity with the anti-MSK1 antibody. Scale bars = 100 μm.

Supplementary Figure S2

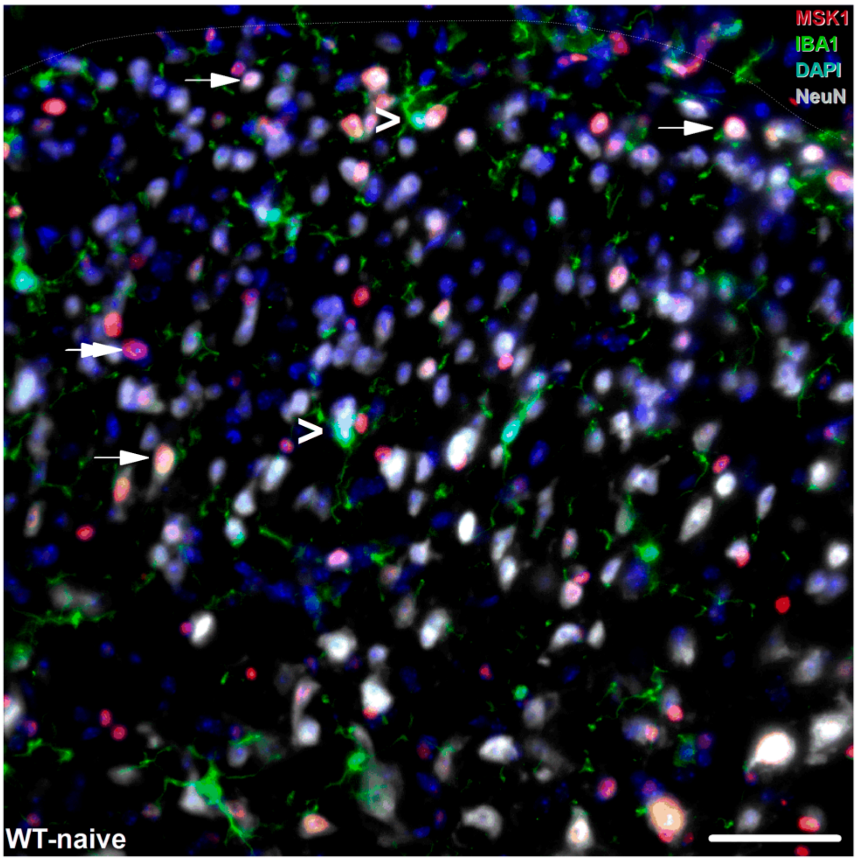

MSK1 expressed in nuclei of sub-populations of neurons and microglia in the mouse spinal dorsal horn.

High magnification microphotograph depicting MSK1-expression in nuclei of neurons (arrow) and microglia (arrowhead). Double arrow indicates a MSK1-expressing nucleus of a presumably oligodendrocyte. Scale bar = 25 µm.

Supplementary Figure S3

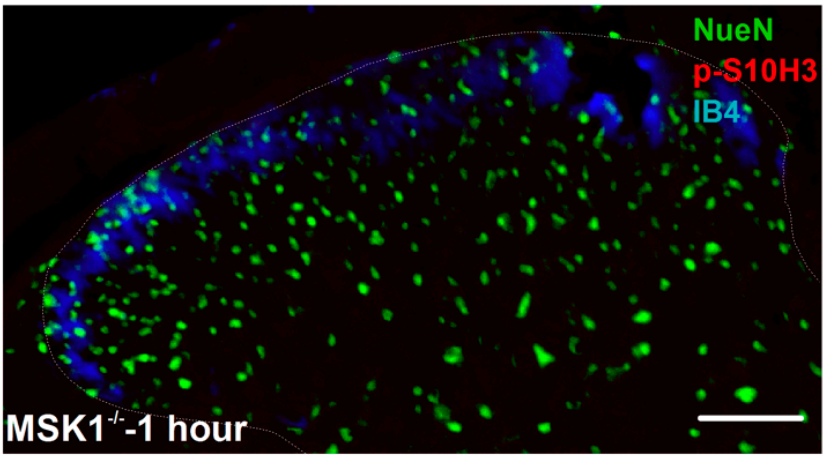

Histone 3 phosphorylation at serine 10 depends on MSK1.

Microphotograph showing no p-S10H3 expressing nuclei in MSK1$^{-/-}$ mice's spinal dorsal horn 1 hour after CFA injection into one of the hind paws. Scale bar = 100 μm.

Supplementary Figure S4

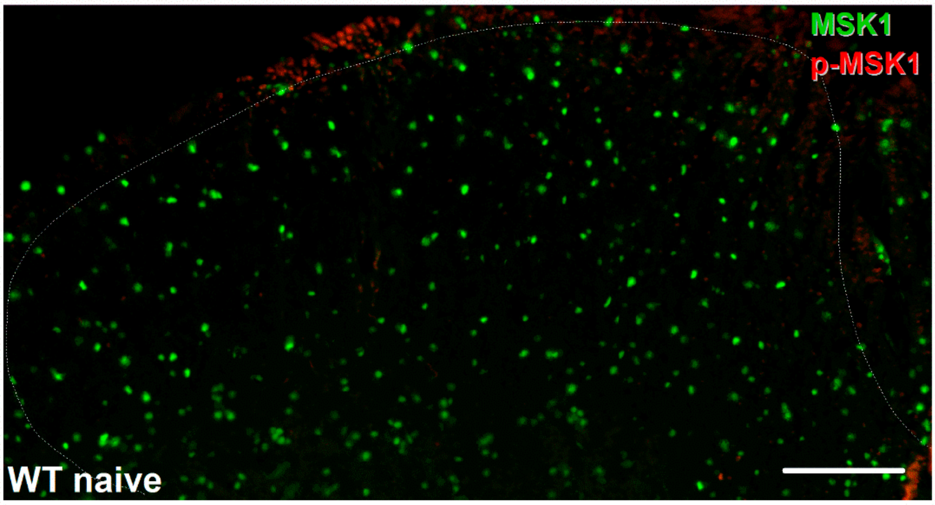

No activated MSK1 is detectable in naive condition in the spinal dorsal horn

Microphotograph showing no p-MSK1-expressing nuclei in naive condition WT mouse spinal dorsal horn. Scale bar = 100 μm.

Supplementary Table S1

	Laminae I-II Absolute	Laminae I-II Relative
**NeuN**		
DAPI	1769	
MSK1	444	25.51±2.64
NeuN	1095	61.02±2.31
MSK1 in NeuN	311	29.1±4.82
NeuN in MSK1		69.88±4.94
**GFAP**		
DAPI	1378	
MSK1	315	23.3±1.56
GFAP	92	7.08±1.06
MSK1 in GFAP	6	6.4±2.51
GFAP in MSK1		2.04±0.73
**IBA1**		
DAPI	1894	
MSK1	539	25.66±4.75
IBA1	186	8.76±1.94
MSK1 in IBA1	96	47.29±4.45
IBA1 in MSK1		17.21±3.54
**CNPase**		
DAPI	1359	
MSK1	333	24.71±0.76
CNPase	28	0.95±0.02
MSK1 in CNPase	13	47.22±7.73
CNPase in MSK1		3.86±0.19
**LMX1b**		
DAPI	1428	
MSK1	344	24.11±0.51
LMX1b	247	17.29±0.68
MSK1 in LMX1b	46	18.67±1.61
LMX1b in MSK1		13.31±0.72
**PAX2**		
DAPI	1312	
MSK1	365	27.72±1.13
PAX2	368	28.47±4.41
MSK1 in PAX2	111	31.3±5.52
PAX2 in MSK1		30.47±1.9

ANTIBODY/REAGENTS	COMPANY	REFERENCE or CATALOG/LOT	HOST SPECIES	CONCEN
**Primary antibodies**				
Anti-CNPase (2',3'-cyclic nucleotide 3'-phosphodiesterase) antibody	antibodies.com	A85409<br>Lot: X23	Chicken	1:2[illegible]
nti-GFAP (glial fibrillary acidic protein) antibody	Abcam	ab53554<br>Lot: GR119165-1	Goat	1:[illegible]
ti-Iba1 (ionized calcium-binding adaptor molecule 1) antibody	Wako Pure Chem	-	Rabbit	1:1[illegible]
Isolectin GS-IB4 biotin-XX conjugate	Invitrogen by Thermo Fisher Scientific	2304295<br>I21414	-	1:1[illegible]
nti-Lmx1b (LIM homeobox transcription factor 1 beta) antibody	Donated by Max Delbrück Centre for Molecular Medicine, Berlin		Guinea pig	1:2[illegible]
nti-MSK1 (mitogen- and stress-activated protein kinase 1) (C27B2) antibody	Cell Signaling Technology	3489S	Rabbit	1:500 (for [illegible]<br>1:1000 (witho[illegible]<br>1:10000 (us[illegible]
Anti-NeuN (neuronal nuclear protein) antibody	Synaptic System	266004	Guinea pig	1:[illegible]
Anti-PAX2 (paired box genes 2) antibody	Invitrogen	Lot: RA230449	Rabbit	1:1[illegible]
Anti-Phospho-Histone H3 (Ser10) antibody	Invitrogen	PA5-17869	Rabbit	1:150 (C[illegible]<br>1:300 (C[illegible]
Anti-Phospho-MSK1 (Thr581) antibody	Cell Signaling Technology	9595(S)	Rabbit	1:[illegible]
**Secondary antibodies**				
finiPure Fab Fragment Goat Anti-Rabbit IgG (H+L)	Jackson ImmunoResearch	111-007-003	Goat	1:[illegible]
lexa Fluor™ 568 donkey anti-rabbit IgG (H+L) red	Invitrogen	A10042<br>Lot: 1826664	-	1:[illegible]
xa Fluor™ 488 donkey anti-rabbit IgG (H+L) green	Invitrogen	A21206	-	1:[illegible]
Alexa Fluor R 488-conjugated AffiniPure F(ab')2 Fragment Donkey Anti-Guinea Pig IgG (H+L)	Jackson ImmunoResearch	706-546-148	Donkey	1:[illegible]
Alexa FluorTM 488 donkey anti-rabbit IgG (H+L)	Invitrogen	A21206	N/A	1:[illegible]
™ 3-conjugated AffiniPure Fragment donkey anti-goat IgG (H+L)	Jackson ImmunoResearch	705-166-147	-	1:[illegible]
Donkey anti-chicken IgY (H+L) FITC	Invitrogen	SA1 – 72000<br>Lot:UC2732211	-	1:[illegible]
C-conjugated AffiniPure donkey anti-guinea pig IgG (H+L)	Jackson ImmunoResearch	706-095-148	-	1:[illegible]
Streptavidin, Alexa Fluor® 350 conjugate	Invitrogen™<br>Molecular Probes™	S11249/449352	-	1:1[illegible]
**Antibody blocking and diluting sera**				
NDS (normal donkey serum)	Abcam	ab 138579<br>Lot: GR3414342-1	-	10% for [illegible]<br>1% for anti[illegible]
NSS (normal sheep serum)	Jackson ImmunoResearch	013-000-121<br>Lot: 158362	-	10% for [illegible]<br>1% for anti[illegible]
**Nuclear staining and antifade agents**				
ProLong™ Gold antifade reagent with DAPI (4',6-diamidino-2-phenylindole)	Invitrogen by Thermo Fisher Scientific (Life Technologies)	P36935<br>2342143	-	
ECTASHIELD Mounting Medium for Fluorescence	Vector Laboratories	ZB0901	-	
